# Physicochemical composition and long-chain fatty acids in fresh and freeze-dried colostrum from cows and buffaloes

**DOI:** 10.3389/fvets.2026.1776116

**Published:** 2026-04-01

**Authors:** Rosy G. Cruz-Monterrosa, J. Efrén Ramírez-Bribiesca, María M. Crosby-Galván, Elsa M. Crosby-Galván, Daniel Mota-Rojas, Ignacio A. Domínguez-Vara, Diana T. Ramírez, Ethel C. García y González

**Affiliations:** 1 Departamento de Ciencias de la Alimentación, División de Ciencias Biológicas y de la Salud, Universidad Autónoma Metropolitana Unidad Lerma, Lerma de Villada, Estado de México, Mexico; 2 Programa de Ganadería, Colegio de Postgraduados-Campus Montecillo, Texcoco, Estado de México, Mexico; 3 Neurofisiología, Comportamiento y Evaluación del Bienestar Animal, DPAA, Universidad Autónoma Metropolitana (UAM), Ciudad de México, Mexico; 4 Facultad de Medicina Veterinaria y Zootecnia, Instituto Literario, Universidad Autónoma del Estado de México, Toluca, Estado de México, Mexico; 5 Department of Chemistry, UC Davis, Davis, CA, United States; 6 Escuela Superior de Medicina Veterinaria y Zootecnia No. 3, Universidad Autónoma de Guerrero (UAGro), Técpan de Galeana, Guerrero, Mexico

**Keywords:** buffalo, colostrum, cow, fatty acids, freeze-dry

## Abstract

**Background:**

Colostrum is a functional food for newborn calves and can be preserved by refrigeration, freezing, or freeze-drying. The physicochemical characteristics of colostrum may or may not be affected by the freeze-drying process. No information is available on freeze-dried buffalo colostrum. The objective of this study was to evaluate the physicochemical quality and fatty acid profile of fresh and freeze-dried buffalo colostrum collected 10–12 h postpartum, and to compare them with those of cow colostrum.

**Methods:**

Colostrum was collected from ranches located in the Mexican highlands. The samples were classified as: (a) fresh cow colostrum (FCC), (b) fresh buffalo colostrum (FBC), (c) 24-h freeze-dried cow colostrum (FdC24), (d) 24-h freeze-dried buffalo colostrum (FdB24), (e) 48-day freeze-dried cow colostrum (FdC48), and (f) 48-day freeze-dried buffalo colostrum (FdB48).

**Results:**

Fat and protein content did not differ between FCC and FBC (*P* > 0.05), while the fat and protein content of FdC24 and FdC48 decreased compared to FdB24 and FdB48 (*P* < 0.05). Lactose, total solids, pH, and IgG did not differ (*P* > 0.05) among groups. NE was higher in buffalo colostrum compared to cow colostrum (*P* < 0.05). SFA in fresh and freeze-dried colostrum were higher (*P* < 0.05) in cow colostrum. MUFA were higher (*P* < 0.05) in buffalo colostrum.

**Conclusion:**

The analysis revealed that saturated fatty acid levels were elevated in colostrum from cows, whereas monounsaturated fatty acids and polyunsaturated fatty acids were more abundant in buffalo colostrum. However, these findings should be substantiated by measurements of lipid oxidation markers, fat globule membrane integrity, and peroxide values. The freeze-drying process tends to reduce fat content and alter the fatty acid profile. Therefore, proper preservation of colostrum is crucial for maintaining its quality; studies indicate that freeze-drying for up to 48 h can effectively preserve its characteristics, making it suitable for use in neonates and other specific nutraceutical applications.

## 1 Introduction

Colostrum is a slightly whitish or yellowish liquid with a viscous consistency; it is formed in the mammary gland during the last third of gestation, and its fluid is secreted approximately 5 days after birth, gradually converting into milk (1). The components of colostrum include immunoglobulins, maternal leukocytes, growth and antimicrobial factors, hormones, cytokines, nutrients, and water. Bovine colostrum contains approximately 1 × 10^6^ cells/ml of maternal leukocytes, mainly macrophages, with 40% to 50% of the colostrum leukocytes; the remainder are lymphocytes and neutrophils. Colostrum leukocytes release cytokines and enhance lymphocyte responses, increasing phagocytosis and the ability to inhibit bacteria; this effect is mediated by stimulation of the humoral immune response (2). Other substances contained in colostrum are lactoferrin, growth hormone, and insulin (3). In particular, insulin-like growth factor type IGF-I modulates gastrointestinal tract development in newborns, increasing intestinal villus size. Immunoglobulin G (IgG) predominates in colostrum and milk; its content ranges from 65% to 90% of total antibodies (4).

Calves at birth are susceptible to developing infectious diseases. Inadequate immunoglobulin G (IgG) intake causes high mortality; therefore, they must consume a sufficient amount of colostrum to maintain an adequate immunoglobulin profile in the first 24 h of life (5). Immunoglobulins and lipid content in colostrum are essential components for offspring to acquire immunity and provide the energy needed to regulate thermoregulation. Maternal colostrum contains 1.9% to 2.3% lipids, which include saturated fatty acids (SFA) and monounsaturated fatty acids (MUFA). Colostrum and breast milk contain triglycerides, the most abundant of which are palmitic, oleic, linoleic, and alpha-linolenic acids. The acids with the lowest proportions are arachidonic, eicosatrienoic, docosahexaenoic, and eicosapentaenoic. Long-chain fatty acids come from the diet, adipose tissue, and liver metabolism, while short and medium-chain fatty acids are synthesized *de novo* within the mammary gland (6). The function of lipids is to produce heat and energy to maintain thermogenesis and fatty acid oxidation. Some fatty acids are beneficial for health due to their antimicrobial action (7).

IgG is a key globular protein, comprising approximately 70% of colostral serum, and plays a crucial role in combating both enteric and respiratory infectious diseases. Buffalo colostrum, in particular, is distinguished by its exceptionally high IgG concentration of 54.0 mg/ml (5). Immunoglobulins belong to a family of glycoproteins that consist of 82%−96% protein and 4%−18% carbohydrates. Previous research has identified two subclasses of IgG in bovine colostrum: IgG1 and IgG2. Furthermore, the elevated levels of non-fucosylated and sialylated oligosaccharides in buffalo colostrum IgG suggest their potential for use in targeted therapies that promote effective anti-inflammatory responses (8). Buffalo colostrum is also characterized by higher concentrations of fats and essential minerals such as calcium, phosphorus, and magnesium compared to cow colostrum (9). A lipidomic analysis has revealed the presence of eight lipids with high expression levels and nine with reduced expression in buffalo colostrum when compared to cow colostrum. Notable components include increased levels of phosphatidylinositol with fatty acid compositions of 18:0/20:2 and 18:0/18:1, as well as phosphatidylethanolamine with a composition of 18:2/22:3. These specific lipids play a vital role in enhancing membrane flexibility and fluidity (10). The unique lipid composition of buffalo colostrum indicates its effectiveness in regulating lipid metabolism and modulating inflammation, making it a preferred option for those with metabolic health concerns or individuals seeking cardiovascular and neuroprotective support (11).

The alternatives for preserving colostrum are refrigeration at −4 °C or freezing at −20 °C. The procedure must be immediate, as both processes maintain colostrum quality for only a short time; after 72 h, bioactive components decrease and the risk of bacterial proliferation increases (12). Another viable alternative is freeze-drying; currently, no information is available on the quality of buffalo colostrum processed this way. In general, freeze-drying is used to preserve heat-sensitive biological materials because processing temperatures are low and the frozen material undergoes a rapid local transition from the hydrated to the dehydrated state, thereby minimizing protein denaturation (13). It is an effective method that preserves immunoglobulin function and total solids recovery; however, studies on fat measurement in colostrum are limited, and attention has not focused on its fatty acid composition. Some background indicates that spray-dried colostrum retained 94% of the immunoglobulin mass (4), but it is unknown whether the freeze-drying time affects lipid quantity and quality. The objective of this study was to compare fresh and freeze-dried cow and buffalo colostrum (24 and 48 days after collection) to evaluate the physicochemical variables, quantity, and integrity of fatty acids.

## 2 Materials and methods

### 2.1 Animals and management

Two ranches in the same production unit of the Mexican highlands were selected, located at a north latitude of 19°0′98" and a west longitude of 99°9′39", with an average annual temperature of 23 °C. The groups of dairy cows (Holstein) and buffaloes (*Bubalus bubalis*) had an average age of 3.5 + 0.3 years at calving, 2–3 births, with weights of 500 to 700 kg under the same management conditions. The animals have a resting area separate from grazing and are fed in stalls twice daily. The forage for animal feed is planted in cultivation areas along the borders of the ranches. Primarily, the ingredients are corn grain and fodder, corn silage, oat-barley straw, and a concentrate formulated at 18% protein. The females are milked for the sale of milk and the elaboration of commercial cheeses. All animals were clinically examined on the day of calving, and those exhibiting any sign of disease or altered colostrum appearance (watery, clots, or blood) were excluded. At the same time, all animals had a body condition score of 3 (on a 1–5 scale).

### 2.2 Collection and experimental distribution of colostrum samples

**Figure 1:**
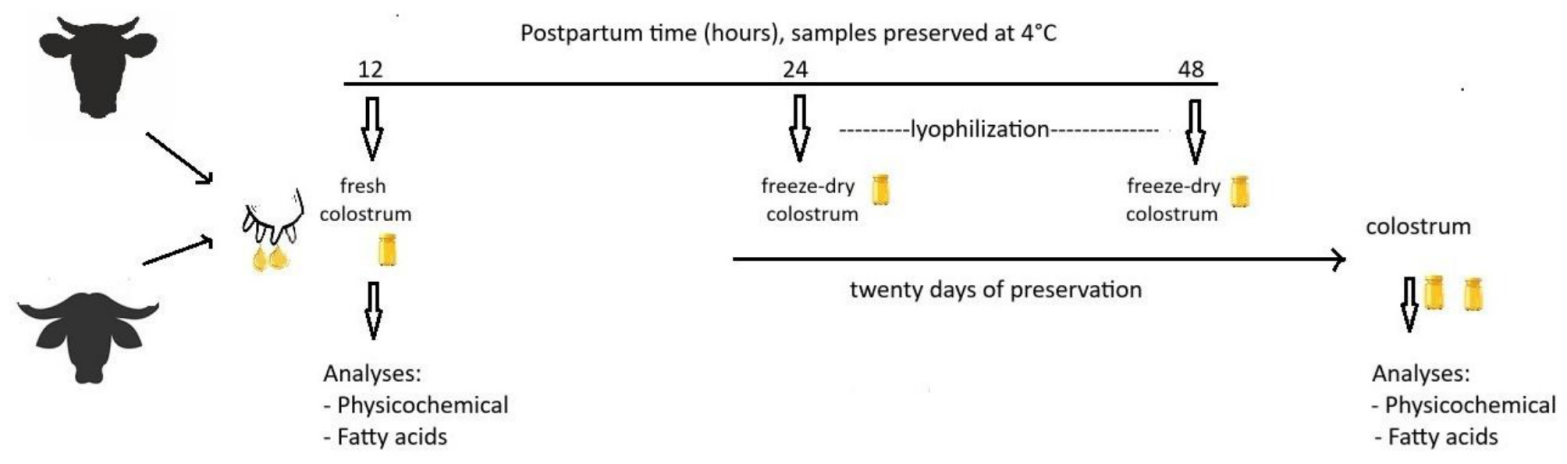
Procedures and times for collecting colostrum from buffalo and cow.

Twenty-six healthy females (13 cows and 13 buffalo) with no history of infections or metabolic diseases were selected from each group. The date and time of calving for each female were recorded. Newborn calves were immediately cared for, with cleaning and disinfection of the umbilical cord; they were then nursed with the first colostrum secretions. Subsequently, the udders were carefully washed with an iodine solution, then dipped and cleaned with cloths. Colostrum was collected between 10 and 12 h after calving from the females during the summer season (August and September). Approximately 600 ml of colostrum was collected from each animal in clean plastic containers. These were transported in coolers at approximately 4 °C to the Postgraduate College laboratory in Montecillo, Mexico, for no more than 90 min. The samples were immediately divided into three equal subsamples (200 ml each) and stored at 4 °C. The first subsamples were classified as: (a) fresh cow colostrum (FCC), and (b) fresh buffalo colostrum (FBC). The second subsamples were kept refrigerated and, after 24 h, were freeze-dried and classified as: (c) 24-h freeze-dried cow colostrum (FdC24), and (d) 24-h freeze-dried buffalo colostrum (FdB24). The third group of subsamples was refrigerated for 48 h and then freeze-dried, classified as: (e) 48-h freeze-dried cow colostrum (FdC48), and (f) 48-h freeze-dried buffalo colostrum (FdB48). Thirteen replicates were collected per treatment, for a total of 78 samples. The physicochemical analyses of the fresh colostrum samples were performed immediately. The freeze-dried samples, dried at 24 and 48 h, were analyzed 20 days after freeze-drying. The experimental procedure is described in Figure 1.

### 2.3 Physicochemical analysis of colostrum

Measurement of colostrum quality: the colostrum dry matter is processed from 20 g of sample at 102 °C for 48 h using the technique described by FSSAI (14). Colostrum analyses were calibrated on the MilkoScan FT120-FOSS using established ranges for fat, total protein, lactose, and total solids. During the analysis, the colostrum samples were taken out of the refrigerator and immersed in warm water at a temperature ranging from 20 °C to 22 °C. Prior to introduction into the MilkoScan, the colostrum was gently homogenized using a vortex mixer due to its dense, high-fat composition. The specific gravity of the colostrum was determined at ~20 °C using a lactometer (23A 38). The pH was measured with a pH meter equipped with a glass electrode (PHEP4, HANNA). Simultaneously, three continuous readings were obtained and displayed on the team, which were photographed and stored in the WhatsApp Web of an iPhone.

IgG measurement: colostrum samples were digested with commercial rennet (0.5 ml of commercial rennet; 250 mg/50 ml of distilled water). Ten milliliters of each colostrum sample were then placed in a 50 ml flask and heated to 37 °C in a water bath. The mixture was subsequently filtered through Whatman #42 filter paper and tested in an Enzyme-linked immunosorbent assay (ELISA) using the commercial Bovine IgG-ELISA ALP kit. This procedure was performed in accordance with the suggestion of Verma et al. (15).

### 2.4 Preparation of freeze-dried colostrum samples

All colostrum samples were collected and refrigerated at 4 °C. After being stored for 24 or 48 h, the samples were removed from the refrigerator, placed in 60 ml amber glass bottles, and frozen at −20 °C for 6 h. Once frozen, the samples were immediately transferred to a freeze dryer (LABCONCO FreeZone 6; Model 7752020, Kansas, USA) operating at 0.080 mBar and −50 °C (16) for 30 h. The freeze-dried samples were stored at −20 °C, and the scheduled analyses were conducted 20 days later.

### 2.5 Analysis of fatty acids

Separation of fat in colostrum: each 25 ml of fresh colostrum was deposited in Nalgen tubes and centrifuged at 7,244.64 g × 30 min at 4 °C in a Beckman J2-HS centrifuge. Subsequently, the fat layer was separated and placed in 1.5- to 2-ml Eppendorf tubes, then centrifuged at 12,281. 23 g × 20 min (EBA21 Hettich Zentrifugen centrifuge), then the upper phase was separated (17).

Fatty acid methylation and esterification process: fresh and freeze-dried colostrum samples (24 and 48 h) were analyzed for the long-chain fatty acid profile, using three methylation techniques (18–20). 0.2 g of fresh colostrum fat and 0.5 g of freeze-dried colostrum from 24 and 48 h were weighed. The transesterification process was carried out in 50 ml culture tubes with a Bakelite lid. 2 ml of 0.5 M methanolic sodium methoxide (Sigma-Aldrich 403067-250ML) was added, mixed by vortexing, and incubated in a water bath. Water at 50 °C for 10 min, then cooled for 5 min.

The acid esterification process in the presence of alcohol was performed by uncovering the tubes and adding 3 ml of 3 M methanolic hydrochloric acid solution (Sigma-Aldrich 33050-U). Again, the tubes were capped, vortexed, incubated at 80 °C for 10 min in a water bath, and then cooled for 7 min. The extraction of methyl ethers was carried out with the addition of 3 ml of hexane and 5 ml of a 6% potassium carbonate solution. The tubes were capped, shaken, and the contents were poured into polypropylene tubes. Subsequently, the samples were centrifuged at 1,232.6 g for 5 min, and the organic phase was collected into polypropylene tubes containing 0.5 g of sodium sulfate and 0.1 g of activated carbon. The tubes were vortexed and centrifuged at 1,232.6 g for 5 min. Finally, the samples were filtered through 0.45 μm Acro discs and placed in amber vials for injection into the chromatograph.

Quantification of fatty acids: the colostrum samples were analyzed in an HP brand gas chromatograph (Hewlett Packard 6890 USA), FID Detector and G2613A automatic injector, silica capillary column (100 m × 0.25 mm × 0.20 μm thick, SPTM-2560, Supelco). Helium (Helium Split ratio 10) was used as carrier gas, the injector and detector temperatures were 250 and 260 °C, respectively. The standard used was FAME Mix C4-C24, 100 mg (Fatty Acids Methyl Esters), catalog number 18,919, SUPELCO. Conjugated linoleic acid (CLA) analysis was calculated using the 9 Cis, trans 11, CLA > 90% 1 g/ml Un-Chek standard. Quantifications of long-chain fatty acids were estimated with Fluka brand linoleic acid with catalog number 62230-5ML-F, oleic acid (Sigma Aldrich, catalog 364525-25mL), stearic acid (Sigma, catalog S-4751) and a gram of palmitic acid (Sigma, catalog P5585-10G), myristic acid (Sigma, catalog M3128-10G), linolenic acid (Fluka, catalog 62160) and 1 ml of arachidonic acid (Sigma, catalog A9673-1G). The samples of each colostrum were analyzed in triplicate, with a difference of < 5% considered significant.

### 2.6 Calculations

The National Research Council (21) equations were used to calculate the metabolizable energy (ME) and net Energy (NE) in colostrum:

ME (Mcal/kg) = 0.057 × protein (%) + 0.092 × fat (%) + 0.0395 × lactose (%) × 0.97 × 0.96 ΣNE (Mcal/kg) = Metabolizable Energy × 0.86

Atherogenecity (AI) and thrombogenicity (TI) colostrum index were calculated with the following formulas (22):


$$\begin{array}{l} AI=\frac{\left[\left(4\;x\;C14:0\right)+C16:0+C18:0\right]\;}{\left[\Sigma MUFA+\;\Sigma PUFAn6+\;\Sigma PUFAn3\right]}\;TI=\frac{\left[C14:0+C16:0+C18:0\right]\;}{\left[0.5\;x\;\Sigma MUFA+\;0.5\;x\;\Sigma PUFAn6+\;3\;x\;\Sigma PUFAn3+\;\Sigma PUFAn3/\Sigma PUFAn6\right]} \end{array}$$

### 2.7 Statistical analysis

A completely randomized design was employed, featuring a factorial arrangement with six treatments and 13 replicates per treatment. Data analysis was conducted using the PROC ANOVA procedure in SAS (version 9.4, SAS Institute Inc., Cary, NC, USA) (23). The statistical model used is expressed as follows:


$$\begin{array}{l} Y\text{ij}=\mu +t\text{i}+e\text{ij} \end{array}$$

In which Y*ij* denotes the response variable for the physicochemical analysis of colostrum, μ represents the population means, t*i* indicates the effect of the treatment, and e*ij* signifies the experimental error. To compare treatment means, Tukey's test was used at the significance level *P* ≤ 0.05.

## 3 Results

### 3.1 Physicochemical levels in fresh and freeze-dried cow and buffalo colostrum

Table 1 shows the chemical composition of fresh and freeze-dried colostrum from cows and buffaloes. The results indicated that the dry matter content of fresh-colostrum from cows and buffaloes was ~27.5 (*P* > 0.05), and freeze-dried from cows and buffaloes was ~97.8 (*P* > 0.05). The fat and protein content did not differ between FCC and FBC (*P* > 0.05), while the fat and protein content of FdC24 and FdC48 decreased (8.4 and 13.5) compared to FdB24 and FdB48 (9.3 and 15.0) (*P* < 0.05). Lactose, total solids, and pH levels did not differ (*P* > 0.05) among species and freeze-drying times, averaging 2.7, 25.75, and 6.61, respectively. Specific gravity was highest in fresh colostrum from both species (1.065), while FdC48 had the lowest level (1.04) (*P* < 0.05). IgG levels did not differ among groups, with a mean of 52.04. ME did not differ between FCC and FBC (*P* > 0.05), although there was a trend toward higher levels in FBC (8 units). ME was higher in FdB24 and FdB48 (1.70) compared to FdC24 and FdC48 (1.53). NE was higher in buffalo colostrum (1.46) compared to cow colostrum (1.34) (*P* < 0.05).

**Table 1:** Physicochemical levels in fresh and freeze-dried cow and buffalo colostrum.

Item	Fresh colostrum		Freeze-dried colostrum			
			24 h		48 h	
	Cow	Buffalo	Cow	Buffalo	Cow	Buffalo
Dry matter, %	26.7 ± 2.31a	28.4 ± 2.34a	97.4 ± 1.43b	98.3 ± 1.24b	97.5 ± 1.31b	98.1 ± 1.56b
Fat. %	9.10 ± 0.11a	9.30 ± 0.12a	8.70 ± 0.10b	9.31 ± 0.13b	8.10 ± 0.11b	9.30 ± 0.14a
Protein, %	14.01 ± 0.16a	15.01 ± 0.13a	13.60 ± 0.19 a	15.01± 0.16a	13.50 ± 0.19a	15.01 ± 0.14a
Lactose, %	2.71 ± 0.08a	2.90 ± 0.09a	2.60 ± 0.06ª	2.90 ± 0.07a	2.50 ± 0.08a	2.80 ± 0.09a
Total solids^1^	25.31 ± 1.2a	26.20 ± 1.01a	25.31 ± 1.03a	26.21 ± 1.01a	25.31 ± 1.03a	26.20 ± 1.08a
Specific gravity	1.06 ± 0.01a	1.07 ± 0.02a	1.05 ± 0.02ab	1.05 ± 0.02ab	1.04 ± 0.03b	1.05 ± 0.04a
pH	6.64 ± 0.11a	6.62 ± 0.12a	6.64 ± 0.14a	6.61 ± 0.13a	6.60 ± 0.14a	6.59 ± 0.13a
IgG (mg/mL)	54.98 ± 5.6a	52.46 ± 6.1a	51.89 ± 7.1a	50.67 ± 6.8a	50.79 ± 6.1a	51.46 ± 5.9a
ME (Mcal/kg)	1.62 ± 0.10a	1.70 ± 0.12a	1.56 ± 0.11b	1.70 ± 0.13a	1.50 ± 0.10b	1.70 ± 0.14a
NE (Mcal/kg)	1.39 ± 0.11a	1.46 ± 0.11b	1.34 ± 0.12a	1.46 ± 0.13b	1.29 ± 0.10a	1.46 ± 0.11b

IgG, inmunoglobulin G; ME, metabolizable energy; NE, net energy. All values are mean ± SEM. The different letters indicate statistical differences at the *P* < 0.05 level of the samples in the same line. ^1^Corroborated with brix refractometry.

### 3.2 Fatty acid levels in fresh and freeze-dried cow and buffalo colostrum

**Figure 2:**
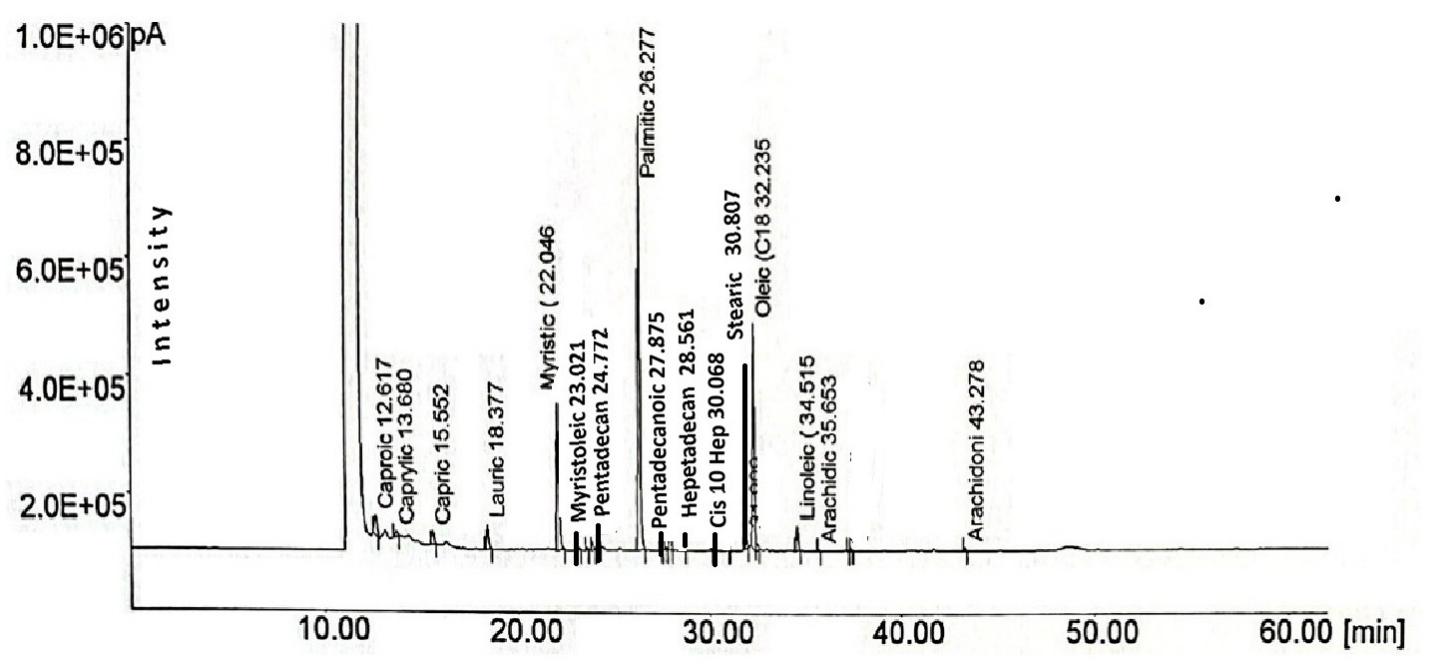
Retention times recorded in the fatty acid chromatogram in colostrum samples.

Figure 2 shows the retention times and areas for each of the fatty acids that were identified and measured in this study, and Table 2 shows the content of fatty acids measured for each gram of total fat. FBC had more concentration (*P* < 0.05) of myristic, myristoleic, palmitoleic, Cis-10 heptadecanoic, and oleic than FCC. On the reverse side, FCC had higher concentrations (*P* < 0.05) of caproic, caprylic, palmitic, stearic, linoleic, and arachidonic acids than FBC. FdC24 and FdC48 had a higher concentration (*P* < 0.05) of caproic, stearic, arachidic, and linoleic acids than FdB24 and FdB48. Conversely, FdB24 and FdB48 had higher concentrations (*P* < 0.05) of myristic, myristoleic, palmitoleic, cis-10 hepta, and oleic acids. Myristic, palmitic, oleic, and linoleic acids decreased in ranges from 2.9% to 18% in the fresh colostrum vs. freeze-dried colostrum; while trans 11 increased by double in fresh colostrum vs. freeze-dried colostrum. Only stearic and arachidonic acids were 1.5 to 3.5 times higher in freeze-dried colostrum than in fresh colostrum. On the other hand, freeze-drying at 24 and 48 h resulted in half the loss of myristic, palmitic, oleic, and linolenic acids in cow colostrum compared to buffalo colostrum; logically, freeze-dried colostrum samples do not have the moisture content of fresh colostrum. The contrast analysis indicates that pentadecanoic acid decreased in FdB48 (0.990) vs. fresh colostrum (1.0085). Pentadecanoic acid, in FdB24, was lower (0.93) than in FdB48 (1.0235). The oleic acid content of FdB24 (22.94) and FdB48h (22.91) was higher (*P* < 0.05) compared to the mean fresh-colostrum (21.86). The cis-9 trans acid content in mean fresh-colostrum (0.264) was higher compared to FdB24 (0.039) and FdB48 h (0.107). Cis-9 trans fatty acid, the mean freeze-dry value at 48 h (0.077) was higher than the mean freeze-dry value at 24 h (0.022). The FdB24 was lower (0.274) compared to the mean of fresh colostrum (0.288). Arachidonic acid in freeze-dried buffalo colostrum at 24 (0.079) and 48 h (0.080) was lower compared to the average of fresh colostrum. CLA (Cis-9 trans) was higher in FBC (0.349) *than in FCC (0.180), and in all freeze-dried colostrum samples* at 24 and 48 h, CLA was lost by about 80%. The AI was higher (15%, *P* < 0.05) in FCC vs. FBC. FbC24 and FbC48 were also higher (8% and 5%, *P* < 0.05) compared to FbB24 and FbB48. The TI was higher in FCC than in FBC (8.8%; *P* < 0.05). FbC24 and FbC48 were also higher (34% and 29%, *P* < 0.05) compared to FbB24 and FbB48.

**Table 2:** Fatty acid levels in fresh and freeze-dried cow and buffalo colostrum.

		Fresh (FC)			Freeze-dried (FdC)					
					24 h			48 h		
		Buffalo (FBC)	Cow (FCC)	SEM	Buffalo (FbB24)	Cow (FbC24)	SEM	Buffalo (FbB48)	Cow (FbC48)	SEM
Saturated fatty acids (SFA)										
Butyric	C 4:0	ND	1.615		ND	2.227		ND	1.912	
Caproic	C 6:0	1.380a	1.850b	0.615	1.584a	2.870b	0.227	1.672a	2.152b	0.046
Caprylic	C 8:0	0.406a	0.516b		0.458a	0.614a		0.460a	0.684b	0.028
Capric	C 10:0	1.102a	1.260a	0.181	1.171a	1.525a	0.348	1.243a	1.630b	0.036
Lauric	C 12:0	2.478a	2.434a	0.456	2.613a	2.781a	0.697	2.597a	2.855b	0.046
Myristic	C 14:0	15.977a	14.056b	0.501	16.284a	14.737b	1.510	16.082a	14.582b	0.617
Pentadecanoic	C 15:0	1.004a	1.013a	0.0085	0.985a	0.875b	0.093	0.990a	1.057b	0.009
Palmitic	C 16:0	37.437a	38.932b	0.124	37.864a	38.792a	1.283	37.927a	38.305a	1.208
Heptadecanoic	C 17:0	0.790a	0.805a	0.033	0.790a	0.733a	0.020	0.814a	0.771a	0.026
Stearic	C 18:0	5.311a	9.123b	0.038	5.429a	8.779b	0.139	5.498a	8.698b	0.045
Arachidic	C 20:0	0.086a	0.152b	0.004	0.084a	0.124b	0.008	0.097a	0.139b	0.001
Monounsaturated fatty acids (MUFA)										
Myristoleic	C 14:1	1.038a	0.715b	0.009	1.036a	0.791b	0.032	1.051a	0.809b	0.013
Palmitoleic	C 16:1	2.769a	2.184b	0.010	2.705a	2.182b	0.031	2.686a	2.245b	0.009
*Cis*-10 Heptadecenoic	C17:1	0.369a	0.280b	0.003	0.364a	0.258b	0.004	0.361a	0.276b	0.006
Oleic	C 18:1 c9	23.323a	20.402b	0.874	22.939a	19.494b	1.179	22.910a	19.532b	1.083
Isomers: C 18	C 18:1	1.116a	1.142a	0.012	1.129a	0.891b	0.031	1.091a	1.259a	0.033
*Cis*-11-Eicosenoic	C 20:1	0.035a	0.031a	0.002	0.034a	0.024a	0.002	0.035a	0.028b	0.002
Polyunsaturated fatty acids (PUFA)										
Linoleic	C 18:2 n6	1.964a	2.530b	0.005	1.906a	2.593b	0.015	1.935a	2.636b	0.011
Linolenic	C 18:3 n3	0.284a	0.292a	0.003	0.274a	0.269a	0.004	0.281a	0.275b	0.003
*Cis*-9 Trans	C 18:2c9 t11	0.349a	0.180b	0.007	0.039a	0.005c	0.001	0.107b	0.048c	0.003
*Cis*-12-trans	C 18:2t10c12	ND	ND		0.030a	ND		0.028a	0.024b	0.002
Arachidonic	C 20:4 n3	0.086a	0.450b	0.003	0.079a	0.352b	0.004	0.080a	0.393b	0.009
Unidentified	–	2.241a	1.652b	0.022	2.206a	1.312b	0.098	2.003a	1.607a	0.160
Atherogenicity (AI) and thrombogenicity (TI) index										
AI		3.203a	3.760b	0.002	3.342a	3.617b	0.001	3.315a	3.492a	0.003
TI		1.894a	2.421b	0.003	1.927a	2.586b	0.002	1.941a	2.508b	0.001

The different letters indicate statistical differences at the *P* < 0.05 level of the samples in the same line. ND, not detected.

Table 2 shows the percentages of fatty acids; FC and LC buffalo colostrum had lower SFA content than FC and LC of cow colostrum at both times (66.8 vs. 70.91, *P* < 0.05), mainly due to higher percentages of palmitic and stearic acids. Specifically, myristic acid was higher in buffalo colostrum vs. cow colostrum (16.11 vs. 14.46). In sequence, the PUFA content in buffalo colostrum was higher than in FC cow colostrum (28.3 vs. 24.15, *P* < 0.05). The PUFA did not show differences (3.98 vs. 3.99, *P* > 0.05) between the colostrum of buffaloes and cows. The highest proportion of PUFA with LC colostrum at 24 and 48 h decreased around 40 and 30% (*P* < 0.05) compared to FC. The differences between the means of LC at 24 and 48 h showed a higher level of C15:0 (10%, *P* < 0.05) in the 48 h LC. Likewise, the PUFA (C18:2 n6, C18:2 c9 t11, C18:2 t10 c12) and the trans showed higher levels with the 48 h LC.

**Figure 3:**
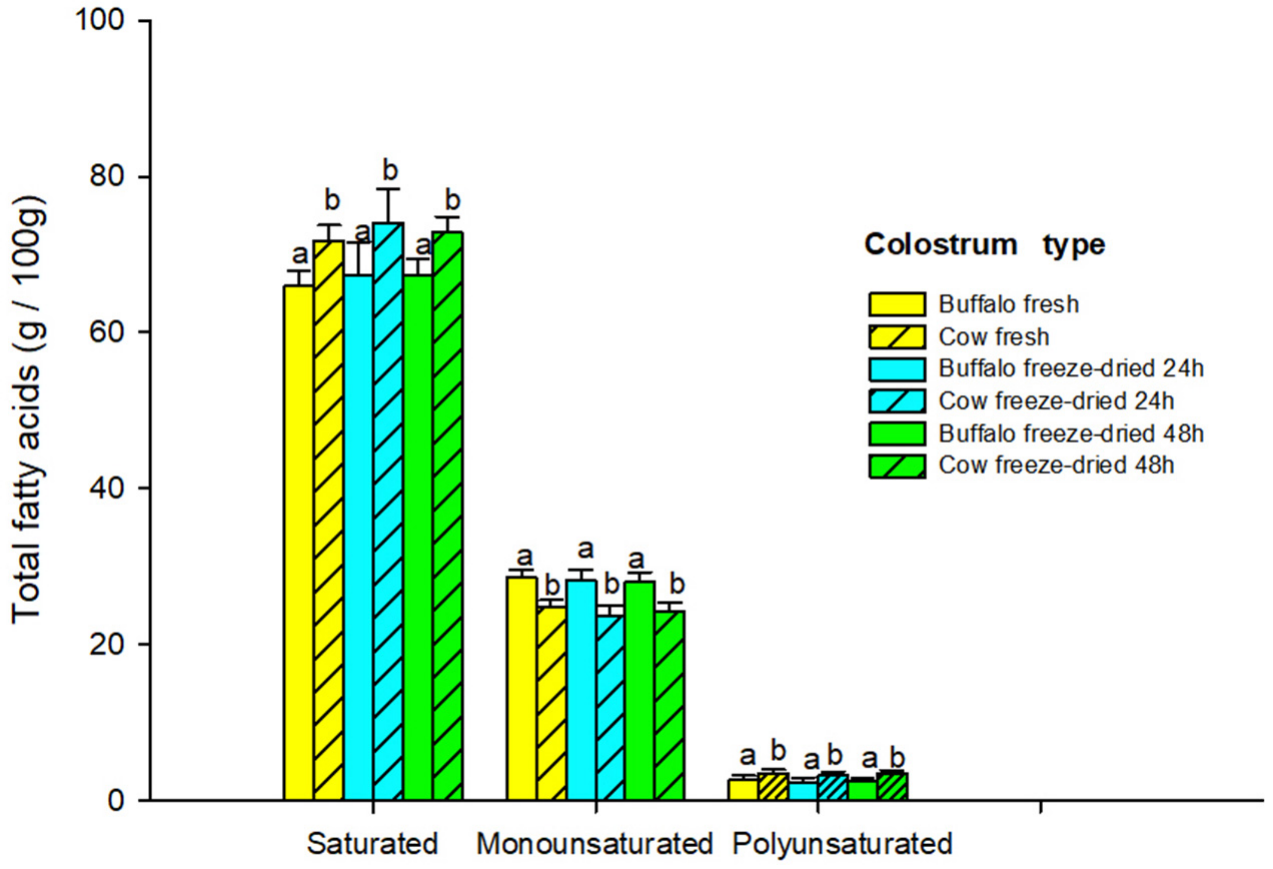
Fatty acid levels in fresh and freeze-dried buffalo and cow colostrum. a, b different letters within each group of colostrum and fatty acid type, indicate a significant difference, *P* < 0.05.

Figure 3 shows the levels of fatty acids by category. SFA in fresh and freeze-dried colostrum at 24 and 48 h were higher (*P* < 0.05) in cow colostrum compared to buffalo colostrum. Conversely, MUFA and PUFA were higher (*P* < 0.05) in buffalo colostrum compared to cow colostrum in both fresh and freeze-dried states at 24 and 48 h.

## 4 Discussion

### 4.1 Physicochemical levels in fresh and freeze-dried cow and buffalo colostrum

In this study, the levels of fat, protein, lactose, total solids, pH, Brix degrees, and IgG did not differ between fresh buffalo and cow colostrum. The total solids and total protein content were similar to those reported in other studies of cattle (24) and buffalo (5). Other studies indicate that the fat content in colostrum is higher in buffalo (between 9.6% and 18.8%) than in cattle (4.60% to 6.7%). Protein values are lower in buffalo, ranging from 5.4% to 13.5%, and in cows, from 12.4% to 14.9%. The total solids content of buffalo colostrum ranged around 24.8%, consistent with another study (25, 26), while the FC samples reached 26.2%. In general, colostrum contains a higher percentage of fat than mature milk; the composition of these fats varies depending on the type of colostrum in different species. O'Callaghan et al. (27) reported that colostrum is richer in palmitic, palmitoleic, and myristic acids than milk. The concentration of colostrum components decreases rapidly during the first 3 days of lactation, except for lactose (27). The yellow color of colostrum is associated with the presence of maternal antibodies, but the Brix scale is more accurate. The Brix scale, measured with a refractometer, correlates with protein concentration, indicating adequate immunoglobulin levels (28). A Brix value of 22% is considered equivalent to 50 mg/ml, indicating high-quality colostrum. In this study, there was a close association between Brix and immunoglobulin concentration in fresh and freeze-dried colostrum, with a Brix value of 22.6% and an equivalent IgG concentration of 52.04 mg/ml, similar to previously reported values. El-Fattah et al. (29) found similar levels of IgG, IgM, IGF-1, and lactoferrin in freeze-dried bovine and buffalo colostrum compared to fresh colostrum. Freeze-drying preserves heat-sensitive biological components because processing temperatures are low and a rapid, local transition of the frozen material from the hydrated to the dehydrated state occurs, thereby minimizing protein denaturation. In this study, the freeze-drying and rehydration process did not affect IgG concentration. On the other hand, protein and lactose levels in cow and buffalo colostrum in this study were similar. Still, other studies report protein and lactose levels of 12% and 2.26%, respectively, in buffalo colostrum (30), and a higher protein level in buffalo colostrum than in cow colostrum (31). The fat level was unaffected in the frozen colostrum, so the reconstituted bovine colostrum showed a decrease in fat content. There is no information on the freeze-drying process, the denaturation of some fatty acids, or the fat loss in the colostrum. It appears that globule size and lipid-derived nutrients, such as fat-soluble vitamins (A and E), are denatured during the freeze-drying process, affecting the total fat level in the colostrum. Other studies using reconstituted freeze-dried equine colostrum did not detect changes in fat composition (32). The decrease in fat in the reconstituted bovine colostrum may indicate a loss of fatty acids during freeze-drying; this is discussed later. In this study, freeze-drying is an effective method for preserving the integrity of various compounds.

### 4.2 Fatty acid levels in fresh and freeze-dried cow and buffalo colostrum

Colostrum contains approximately 400 different fatty acids; PUFAs, such as lauric acid, may have antimicrobial activity (33). Furthermore, the nutritional and metabolic effects of the highly concentrated fatty acids in colostrum provide health benefits for calves. It is important to consider colostrum preservation during the first 3 days; for example, no differences were found between Egyptian buffalo and cow colostrum in the first few days after calving (9). The SFA profile of colostrum differs between primiparous and multiparous animals, reflecting differences in energy status at the start of lactation (34). The major fatty acids in colostrum are myristic, palmitic, and oleic acids, while the lowest values are myristoleic, pentadecanoic, and heptadecanoic acids. Cholesterol concentration ranges from 12.8 mg/100 ml. Short-chain fatty acids (C4 to C12) predominate in buffalo colostrum and lyophilized camel milk (9, 35), although their amounts are very small compared to those in cow colostrum (36). However, the percentages of myristic, palmitic, stearic, and oleic acids are higher. Buffalo FC had a higher amount of linolenic acid, similar to fresh and freeze-dried camel milk (36, 37), and presented higher amounts of linolenic acid and PUFAs compared to cow milk. For both types of milk, the main fatty acids were palmitic, oleic, myristic, and stearic acids. Ibrahim et al. (38) reported higher levels of PUFAs in Egyptian buffalo milk than in Egyptian cow milk. Buffalo milk contains lower amounts of medium-chain fatty acids (C8:0 to C12:0) (31). Buffalo colostrum contains more myristic and palmitic acids and less stearic acid than cow colostrum. Buffalo colostrum also contains a higher amount of rumenic acid (C18:2 c9 tr11, the main CLA than cow colostrum (5, 39). Physiologically, oleic isomer fatty acids are known to be precursors of linoleic isomers, formed in the mammary gland by delta-9 desaturase (40). Varrichio et al. (41) reported that average CLA content was higher in buffalo milk than in cow milk, and a positive correlation was observed between oleic isomer and CLA content in buffalo milk from Mediterranean buffalo (42). Total trans fatty acids (C18:1 trans + C18:2 c9 tr11) were higher in buffalo milk than in cow milk, and PUFAs are primarily associated with the milk fat globule membrane (43), and their content decreases as the milk fat globule membrane size increases. In this study, SFA were found to be more abundant in bovine FC and LC, with stearic acid content in all samples. In contrast, MUFA and PUFA were predominantly present in buffalo colostrum, with linoleic and linolenic acids being the most abundant. These findings warrant further validation through additional physicochemical tests, such as lipid oxidation markers, peroxide value, and fat globule size. Although these tests were not conducted in this study, they should be incorporated into future research to substantiate the observed differences in fatty acid composition.

The AI and TI indices indicate the effects of fatty acids on human health and the likelihood of atherosclerosis, blood clots, and thrombus formation (44). The AI index reflects the association between fatty acids (lauric, myristic, and palmitic) and their classification as proatherogenic or antiatherogenic. Atherogenicity is also associated with inflammatory pathways and the innate immune system (44, 45). The TI index, on the other hand, indicates the tendency for clot formation in blood vessels. Generally, AI and TI are used to assess the effects of different fatty acids in food products on cardiovascular disease (46). No information is available on the AI-TI in colostrum. For milk, reported values have been obtained from sheep milk (AI: 4.21; TI: 2.30 to 3.03), goat milk (AI: 3.17), and cow milk (AI: 3.31; TI: 3.59) (46, 47). This study shows that buffalo colostrum exhibited greater stability in both AI and TI than cow colostrum. The AI and TI indices of buffalo colostrum were lower. Therefore, the use of buffalo colostrum may be more readily accepted, primarily due to its cholesterol-lowering effect, and this stability is maintained in both fresh and freeze-dried colostrum.

## 5 Conclusion

Buffalo are the second most important species for milk production globally, after cattle. This study demonstrated that the quality of fresh and freeze-dried buffalo colostrum surpassed that of cow colostrum. Although cows had a higher level of biologically saturated fatty acids, buffalo exhibited elevated levels of monounsaturated fatty acids and polyunsaturated fatty acids. Specifically, buffalo colostrum contained greater concentrations of palmitic acid, trans fatty acids, linolenic acid, and conjugated linolenic acid compared to that of cows. However, to verify this trend, it is crucial to assess oxidative markers and peroxide values to gauge lipid degradation. Generally, the freeze-drying process can reduce fat content and alter the fatty acid profile; nonetheless, it preserves the chemical and nutritional quality of colostrum. Proper preservation is vital to maintaining colostrum quality, and freeze-drying for up to 48 h has been shown to be effective, making it suitable for neonates and other specialized nutraceutical applications.

## Data Availability

The original contributions presented in the study are included in the article/supplementary material, further inquiries can be directed to the corresponding authors.

## References

[1] McGrath BA Fox PF McSweeney PLH Kelly AL. Composition and properties of bovine colostrum: a review. Dairy Sci Technol. (2016) 96:133–58. doi: 10.1007/s13594-015-0258-x

[2] Puppel K Gołebiewski M Grodkowski G. Composition and factors affecting quality of bovine colostrum: a review. Animals. (2019) 9:1070. doi: 10.3390/ani9121070 31810335 PMC6940821

[3] Alnadari F Ibeogu IH Shoura HE Wang R Yar MS. Immunomodulatory potential of bovine colostrum: a holistic perspective on health, disease resistance, and aging. Anim Adv. (2025) 2:e003. doi: 10.48130/animadv-0025-0001

[4] Baker PH Stafford LS Lange SN. Advancing protective effects of maternal antibodies in neonates through animal models. J Immun. (2025) 214:2523–34. doi: 10.1093/jimmun/vkaf235 41169230

[5] Lotito D Pacifico E Matuozzo S Musco N Lommelli P Zicarelli F . Colostrum composition, characteristics and management for buffalo calves: a review. Vet Sci. (2023) 10:358. doi: 10.3390/vetsci10050358 37235441 PMC10222353

[6] Berenhauser AC Pinheiro do Prado AC da Silva RC Gioielli LA Block JM. Fatty acid composition in preterm and term breast milk. Int J Food Sci Nutr. (2012) 63:318–25. doi: 10.3109/09637486.2011.627843 22023571

[7] Klopp RN Ferreira CR Casey TM Boerman JP. Relationship of Cow and Calf Circulating Lipidomes with Colostrum Lipid Composition and Metabolic Status of the Cow. J Dairy Sci. (2022) 105:1768–87. doi: 10.3168/jds.2021-21008 34802733

[8] Eker F Akdaşçi E Duman H Yalçintaş YM Canbolat AA Kalkan AE . Antimicrobial properties of colostrum and milk. Antibiotics. (2024) 13:251. doi: 10.3390/antibiotics13030251 38534686 PMC10967647

[9] Abd AM El –Fattah AR EL-Dieb M El-Kashef HA. Changes in composition of colostrum of Egyptian buffaloes and Holstein cows. composition and yield of colostrum and milk from Murrah and “Murrah x Carabao” crosses in the Philippines. Trop Anim Sci J. (2021) 44:347–55. doi: 10.1186/1746-6148-8-19

[10] Bhanu MS Amano M Nishimura S-I Aparna HS. Glycome characterization of immunoglobulin G from buffalo (Bubalus bubalis) colostrum. Glycoconj J. (2015) 32:625–34. doi: 10.1007/s10719-015-9608-4 26239923

[11] Li R Wang Y Li C Huang J Zeng Q Li L . Characterization and comparison of lipids in yak colostrum, buffalo colostrum, and cow colostrum based on UHPLC-QTOF-MS lipidomics. Dairy. (2025) 6:14. doi: 10.3390/dairy6020014

[12] Šlosárková SS Pechová A Staněk S Fleischer P Zouharová M Nejedlá E. Microbial contamination of harvested colostrum on Czech dairy farms. J Dairy Sci. (2021) 104:11047–58. doi: 10.3168/jds.2020-19949 34253366

[13] Merivaara A Zini J Koivunotko E Valkonen S Korhonen O. Preservation of biomaterials and cells by freeze-drying: change of paradigm. J Control Release. (2021) 336:480–98. doi: 10.1016/j.jconrel.2021.06.042 34214597

[14] FSSAI. Manual of Methods of Analysis of Foods Milk and Milk Products. New Delhi: Food Safety and Standards Authority of India (Ministry of Health and Family Welfare) (2016)

[15] Verma UK Kumar S Ghosh AK Kumar S Barwal RS Sahi BN. Determination of immunoglobulin G (IgG) concentration in buffalo colostrum and serum of new born calves by indirect ELISA. J Pharmacogn Phytochem. (2018) 7:1233–5.

[16] Oddone I Arsiccio A Duru C Malik K Ferguson J Pisano R . Vacuum-induced surface freezing for the freeze-drying of the human growth hormone: how does nucleation control affect protein stability? J Pharm Sci. (2020) 109:254–63. doi: 10.1016/j.xphs.2019.04.014 31002810

[17] Luna P Juárez M De la Fuente MA. Validation of a rapid milk fat separation method to determine the fatty acid profile by gas chromatography. J Dairy Sci. (2025) 88:3377–81. doi: 10.3168/jds.S0022-0302(05)73021-6 16162510

[18] Sukhija PS Palmquist DL. Rapid method for determination of total fatty acid content and composition of feedstuffs and feces. J Agric Food Chem. (1998) 36:1202–6. doi: 10.1021/jf00084a019

[19] Jenkins TC. Technical note: common analytical errors yielding inaccurate results during analysis of fatty acids in feed and digesta samples. J Dairy Sci. (2010) 93:1170–4. doi: 10.3168/jds.2009-2509 20172237

[20] Palmquist DL Jenkins TC. Challenges with fats and fatty acid methods. J Anim Sci. (2003) 81:3250–4. doi: 10.2527/2003.81123250x 14677882

[21] NRC. Nutrient Requirements of Dairy Cattle. 7th ed. Washington, DC: The National Academies Press (2001) 38386771

[22] Paszczyk B Łuczyńska J. The comparison of fatty acid composition and lipid quality indices in hard cow, sheep, and goat cheeses. Foods. (2020) 9:1667. doi: 10.3390/foods9111667 33203107 PMC7696827

[23] SAS Institute Inc. SAS Version 9.4. Cary, NC: SAS Institute Inc (2019).

[24] Kehoe SI Jayarao BM Heinrichs AJ. A survey of bovine colostrum composition and colostrum management practices on Pennsylvania dairy farms. J Dairy Sci. (2007) 90:4108–16. doi: 10.3168/jds.2007-0040 17699028

[25] Nawar MA. Antimicrobial factors in buffalo milk: changes during the first month of lactation. Egyptian J Dairy Sci. (2006) 34:133–8.

[26] An Z Luo G Gao S Zhang X Chen C Yao Z . Evaluation of parity effect on characteristics and minerals in buffalo (Bubalus Bubalis) colostrum and mature milk. Foods. (2023) 12:1321. doi: 10.3390/foods12061321 36981245 PMC10048450

[27] O'Callaghan TF O'Donovan M Murphy JP Sugrue K Mannion D McCarthy WP . Evolution of the bovine milk fatty acid profile - from colostrum to milk five days post parturition. Int Dairy J. (2020) 104:104655. doi: 10.1016/j.idairyj.2020.104655

[28] Sockett D Breuer RM Smith LW Keuler NS Earleywine T. Investigation of brix refractometry for estimating bovine colostrum immunoglobulin G concentration. Front Vet Sci. (2023) 10:1240227. doi: 10.3389/fvets.2023.1240227 37818390 PMC10560986

[29] El-Fattah A Alaa M Abd Rabo FH EL-Dieb SM El-Kashef HA. Changes in composition of colostrum of Egyptian buffaloes and Holstein cows. BMC Vet Res. (2012) 8:19. doi: 10.1186/1746-6148-8-19 22390895 PMC3344693

[30] Bondoc OL Almendral-Saludesb T Tandangb AG Bustosc AR Ramosa AR Ebrona AO. Composition and yield of colostrum and milk from Murrah and “Murrah x Carabao” crosses in the Philippines. Trop Anim Sci J. (2021) 44:347–55. doi: 10.5398/tasj.2021.44.3.347

[31] Bondoc OL Ramos AR. Fatty acid composition and nutritional indices/ratios of colostrum and milk from Murrah and “Murrah × Carabao” crossbred buffaloes. Philip J Sci. (2022) 151:139–52. doi: 10.56899/151.01.10

[32] de Lima TC de Sobral GG de França Queiroz AES Chinelate GCB Porto TS Oliveira JTC . Characterization of Lyophilized Equine Colostrum. J Equine Vet Sci. (2024) 132:104975. doi: 10.1016/j.jevs.2023.104975 38040068

[33] Amr M Farid A. Impact of cow, buffalo, goat or camel milk consumption on oxidative stress, inflammation and immune response post weaning time. Sci Rep. (2024) 30:9967. doi: 10.1038/s41598-024-59959-8 38693190 PMC11063178

[34] Contarini G Povolo M Pelizzola V Monti L Bruni A Passolungo L . Bovine colostrum: changes in lipid constituents in the first 5 days after parturition. J Dairy Sci. (2014) 97:5065–72. doi: 10.3168/jds.2013-7517 24931528

[35] An Z Wei K Yao Z Yang L Wang C. Fatty acid composition comparison between colostrum and mature milk in buffaloes. Int J Dairy Technol. (2024) 77:1244–9. doi: 10.1111/1471-0307.13122

[36] Gorban AMS Izzeldin OM. Fatty acids and lipids of camel milk and colostrum, *Int J Food Sci Nutr*. (2001) 52:283–7. doi: 10.1080/713671778 11400477

[37] Abu-Lehia IH ALMohizea IS El Beheri M. Physical and chemical characteristics of camel colostrum. Austr J Dairy Technol. (1989) 44*:* 34–6.

[38] Ibrahim AS Zahran HA Awaad SS Hegab OW. Comparative evaluation of fatty acid profiles and lipid nutritional indexes in Egyptian fresh cow, buffalo, goat soft cheeses and their mixtures. Egypt J Chem. (2023) 66:415–24. doi: 10.21608/ejchem.2023.206528.7879

[39] Coroian A Erler S Matea CT Mireşan V Răducu C Bele C . Seasonal changes of buffalo colostrum: physicochemical parameters, fatty acids and cholesterol variation. Chem Cent J. (2013) 7:40. doi: 10.1186/1752-153X-7-40 23442377 PMC3604957

[40] Fernandes SAA Mattos WRS Matarazzo SV Tonhati H Gama S Duarte LP. Activity of Δ^9^-desaturase enzyme in mammary gland of lactating buffaloes. Italian J Anim Sci. (2007) 6:1163–66. doi: 10.4081/ijas.2007.s2.1060

[41] Varricchio ML Di Francia A Masucci F Romano R Proto V. Fatty acid composition of Mediterranean buffalo milk fat. Italian J Anim Sci. (2007) 6(supp 1):509–11. doi: 10.4081/ijas.2007.1s.509

[42] Pegolo S Stocco G Mele M Schiavon S Bittante G Cecchinato A. Factors affecting variations in the detailed fatty acid profile of Mediterranean buffalo milk determined by 2-dimensional gas chromatography. J Dairy Sci. (2017) 100:2564–76. doi: 10.3168/jds.2016-11696 28189314

[43] Couvreur S Hurtaud C. Relationships between milks differentiated on native milk fat globule characteristics and fat, protein and calcium compositions. Animal. (2017) 11:507–18. doi: 10.1017/S1751731116001646 27485694

[44] Rizzo FA Júnior JS Scheibler RB Fluck AC de Vargas DP Nörnberg JL . Biofortification of cow milk through dietary supplementation with sunflower oil: fatty acid profile, atherogenicity, and thrombogenic index. Trop Anim Health Prod. (2023) 55:269. doi: 10.1007/s11250-023-03670-9 37452970

[45] Garaffo MA Vassallo-Agius R Nengas Y Lembo E Rando R Maisano R . Fatty acids profile, atherogenic (IA) and thrombogenic (IT) health lipid indices, of raw roe of blue fin Tuna (Thunnus thynnus L) and Their Salted Product “Bottarga”. Food Nutr Sci. (2011) 2:736–43. doi: 10.4236/fns.2011.27101

[46] Vala AB Dharaiya CN Mehta BN. Differences in milk fat composition across selected mammals - a review. Grasas Aceites (2024) 4:2229. doi: 10.3989/gya.1200232.2229

[47] Pretorius B Schonfeldt H. Cholesterol, fatty acids profile and the indices of atherogenicity and thrombogenicity of raw lamb and mutton offal. Food Chem. (2020) 345:128868. doi: 10.1016/j.foodchem.2020.128868 33352404

